# Development of Prodrugs for PDT-Based Combination Therapy Using a Singlet-Oxygen-Sensitive Linker and Quantitative Systems Pharmacology

**DOI:** 10.3390/jcm8122198

**Published:** 2019-12-13

**Authors:** Luong Nguyen, Mengjie Li, Sukyung Woo, Youngjae You

**Affiliations:** 1 Department of Pharmaceutical Sciences, College of Pharmacy, University of Oklahoma Health Sciences Center, Oklahoma City, OK 73117, USA; Luong-Nguyen@ouhsc.edu (L.N.); Mengjie-Li@ouhsc.edu (M.L.); sukyung-woo@ouhsc.edu (S.W.); 2 Department of Pharmaceutical Sciences, University at Buffalo, State University of New York, Buffalo, NY 14214, USA

**Keywords:** photodynamic therapy, PDT, combination therapy, light-activatable prodrugs, singlet-oxygen-sensitive linker, aminoacrylate, paclitaxel, SN-38, Combretastatin A-4, quantitative systems pharmacology, pharmacokinetics, physiologically based pharmacokinetic modeling

## Abstract

Photodynamic therapy (PDT) has become an effective treatment for certain types of solid tumors. The combination of PDT with other therapies has been extensively investigated in recent years to improve its effectiveness and expand its applications. This focused review summarizes the development of a prodrug system in which anticancer drugs are activated locally at tumor sites during PDT treatment. The development of a singlet-oxygen-sensitive linker that can be conveniently conjugated to various drugs and efficiently cleaved to release intact drugs is recapitulated. The initial design of prodrugs, preliminary efficacy evaluation, pharmacokinetics study, and optimization using quantitative systems pharmacology is discussed. Current treatment optimization in animal models using physiologically based a pharmacokinetic (PBPK) modeling approach is also explored.

## 1. Introduction

Photodynamic therapy (PDT) is a clinically approved treatment for a number of local cancers or local precancerous conditions, e.g., cancer of the esophagus, Barrett’s esophagus, endobronchial cancer, and actinic keratosis, and is currently under investigation to treat many others, including cancers of the skin, brain, mouth, stomach, prostate, cervix, and vagina [1,2]. A major mechanism by which PDT eradicates tumors is by locally generating a reactive oxygen species, i.e., singlet oxygen, which causes cellular damage leading to cell death. Singlet oxygen, however, has a short lifetime and can only diffuse a short distance (approximately 10–320 ns, 10–55 nm in cells) after it is generated, which leads to major, highly site-specific damage produced by PDT [3,4,5,6,7,8,9,10,11]. Given the highly local effect of PDT and the fact that incomplete ablation and recurrence are problems in PDT, partly resulting from residues of surviving cancer cells that escape PDT treatment, there has been a special interest in developing combination treatments involving PDT to make PDT more effective and expand its scope of applications [12,13,14,15,16,17,18,19,20,21]. In particular, the combination of PDT and systemically administered anticancer drugs has been extensively explored to achieve additive or synergistic antitumor effects [19,22,23,24,25]. More recent approaches have been designed to minimize systemic adverse effects of anticancer drugs based on various innovative ideas [12,20,26,27]. Reactive oxygen species were also used for triggering drug release from stimuli-responsive drug-releasing systems [28,29,30,31,32]. In our lab, we have been developing such prodrug systems in which the anticancer drug is released from the inert prodrug specifically at the tumor site when PDT treatment is initiated. The drug provides a sustained and extended cell-killing effect, in addition to local damage produced by PDT. While there are a number of other reviews about prodrugs and PDT [9,16,33,34,35,36,37,38], this case review summarizes the singlet-oxygen-activatable prodrug system developed in our lab, specifically focusing on development of our unique cleavable linker triggered by singlet oxygen, initial evaluation of photodynamic prodrugs utilizing the singlet-oxygen-sensitive linker, quantitative systems pharmacologic analysis, and current optimization aided by physiologically based pharmacokinetic modeling in animal models.

## 2. Development of Singlet-Oxygen-Sensitive Linker

**Figure 1:**
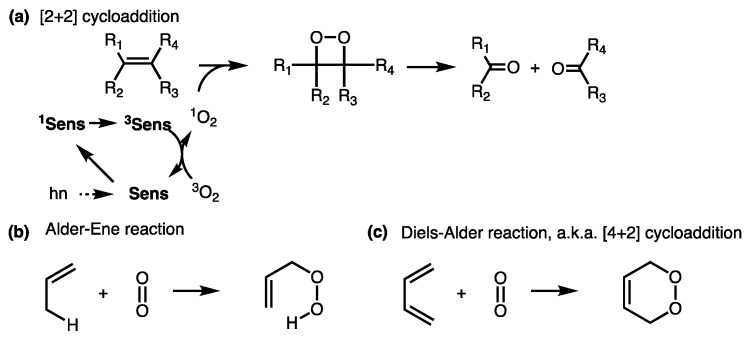
(**a**) [2+2] cycloaddition of singlet oxygen to a double bond to form dioxetane intermediate, followed by a bond breakage; (**b**, **c**) two other reactions competing with the [2+2] cycloaddition; sens = photosensitizer, R_1–4_ represent various substituents.

Photocleavable bonds have long been investigated and used in numerous applications, such as in organic synthesis, drug delivery, and the study of intracellular biochemical processes (e.g., signaling pathways, gene expression) [39,40,41,42,43,44,45]. Light is a major component of PDT; however, the preferred wavelengths—to facilitate deeper penetration in tissue—are in the red and far-red regions, limiting the choices of linkers that are directly cleaved by such low-energy light [44,45,46,47]. In our prodrug system, we exploited a unique bond cleavage phenomenon mediated by singlet oxygen, which is generated during the PDT process, to overcome this problem. The cleavage mechanism is based on a well-known property of the double bond to participate in a [2+2] cycloaddition with singlet oxygen following decomposition to ultimately give carbonyl fragments via a dioxetane intermediate (Figure 1a) [48,49,50,51,52,53,54,55,56].

**Figure 2:**
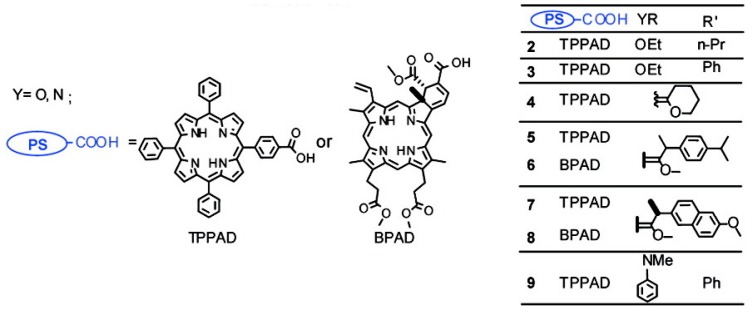
A singlet-oxygen-activatable prodrug system for carbonyl-bearing drugs utilizing a singlet-oxygen-sensitive linker designed by the Dolphin group. Structures of photosensitizer and drugs investigated. Reproduced with permission from [57]. Note: In 5 and 6, R’ was ibuprofen moiety. Me = methyl group (CH_3−_), Et = ethyl group (CH_3_CH_2−_), n-Pr = n-propyl group (CH_3_CH_2_CH_2−_), and Ph = Phenyl group (C_6_H_5−_).

Such a cleavage mechanism has been thoroughly investigated and utilized by a number of investigators to, for example, prepare carbonyl-containing compounds in organic synthesis [54], create prodrugs from drugs bearing a carbonyl group [57], release drugs from liposomes [58,59,60], release and precipitate photosensitizers at targeted sites [61], or deliver photosensitizers from a glass tip [62]. While the [2+2] cycloaddition reactions of double bonds to singlet oxygen are either forbidden or unfavorably compete with Alder–Ene reaction and Diels–Alder [4+2] cycloaddition (Figure 1b,c), certain substitutions around the double bond were shown to facilitate the [2+2] reaction. Data from numerous reports showed that LUMO–HOMO interaction between singlet oxygen and alkene, the configuration around a double bond, and the availability of the hydrogen at the allyl position to the double bond play important roles in directing the reaction between singlet oxygen and an olefin. The reaction of singlet oxygen and an alkene can be exclusively directed through [2+2] cycloaddition, leading to clean products from decomposition of the dioxetane intermediate [50,51,55,63]. For example, various *cis/trans*-substituted alkenes were reported to react with singlet oxygen, followed by decomposition to give clean corresponding carbonyl compounds [49,57,64,65]. When the substituted double bonds were used as a photocleavable linker, a light-triggered release of greater than 90% of the conjugated reagents was reported, i.e., in [54,57,64,65]. Figure 2 represents an example of elegant design to create such activatable prodrugs invented and prepared from the Dolphin group [57]. 

**Figure 3:**
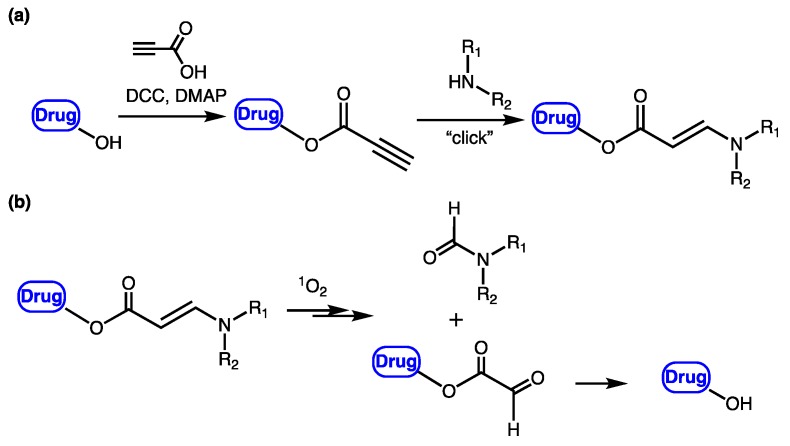
(**a**) Facile preparation of prodrug bearing a β-aminoacrylate linker from a hydroxyl-containing drug; (**b**) Activation of the prodrug and regeneration of original drug by singlet oxygen. DCC = dicyclohexyl carbodiimide, DMAP = 4-(dimethylamino) pyridine, R_1_ and R_2_ = alkyl groups.

In an effort to search for a linker that offered facile conjugation conditions and released intact conjugated drugs, instead of drugs retaining a carbonyl group generated in linkers based on substituted double bonds (Figure 1a and Figure 2), our lab turned attention to an acrylate linker bearing a heteroatom at β position. The investigation led to the discovery of an optimal β-aminoacrylate linker, which was shown to be stable for at least seven days in medium and without light when it was conjugated to a photosensitizer. Incorporation of the linker in drugs bearing an OH group was achieved under mild conditions (typically from 0 °C to room temperature in an aprotic solvent) and with high yield reactions (>80%) (Figure 3a). Cycloaddition with singlet oxygen and cleavage of the linker was fast and efficient. For example, more than 90% of a model prodrug of estrone was cleaved in 10 min (Table 1, entry 1) with a suitable photosensitizer and light source. Alternatively, using coumarin as a fluorescent probe, an almost quantitative release of the conjugated reagent was reported. Notably, intact original drugs/model agents bearing free OH groups were detected by gas chromatography/mass spectrometry after photolysis without the need of a hydrolase when using the β-aminoacrylate linker [66,67] (Figure 3b). The linker was used as a core component of all our subsequent prodrug designs and is referred to interchangeably as an aminoacrylate linker or singlet-oxygen-sensitive linker.

## 3. Design of β-Aminoacrylate-Containing Prodrugs and Initial Biological Evaluation

**Figure 4:**
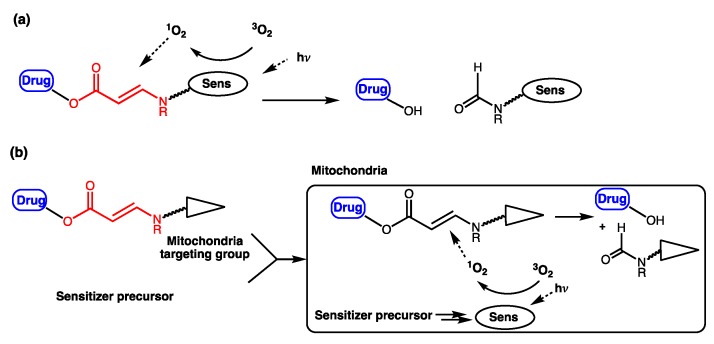
(**a**) Unimolecular design; (**b**) bimolecular design and release of drug upon activation during photodynamic therapy (PDT). Sens = photosensitizer.

One model drug (estrone), three anticancer drugs (SN-38, combretastatin A-4 (CA4), paclitaxel (PTX)), and a number of fluorescent probes were investigated in our lab as prodrugs and proimaging/probing agents activated by PDT using the β-aminoacrylate linker above (Table 1). The drugs contained either a phenolic or an alcoholic hydroxyl group, and they were conjugated to a β-aminoacrylate linker with an ester bond (structures in Table 1). Photosensitizers (PS) were either directly linked to prodrug/proagent molecules (Figure 4a, Table 1, entries 1–8, 11) or chemically separated from the drug moiety, but were localized to specific sites inside cells to which the prodrugs/proagents were also targeted (Figure 4b and Table 1, entries 9, 10, and 12).

Initial testing showed a significant reduction in cytotoxicity (4.8- and 14.5-fold respectively, as measured by toxicity IC_50_ against MCF-7 cells) of SN-38 and CA4 once the drugs were conjugated to a photosensitizer through the β-aminoacrylate linker (Table 1, entries 2 and 3). The toxicity was recovered to a level comparable to that of the original drug upon activation of the prodrug (toxicity IC_50_ against MCF-7 cells was 218 nM and 13 nM, compared with 170 nM and 8 nM of SN-38 and CA4, respectively) (Table 1) [67].

**Figure 5:**
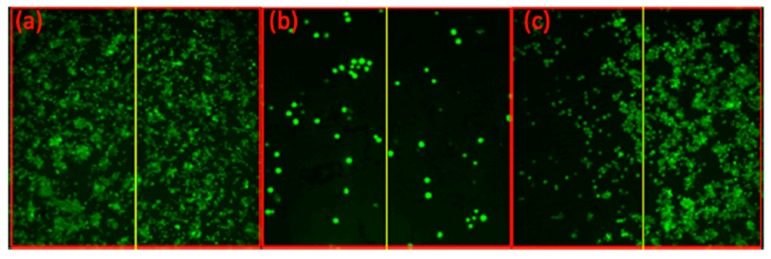
Extended cell-killing effect of the prodrug-PDT system, compared with PDT alone, demonstrated by fluorescence live cell imaging. (**a**) MCF-7 cells control, (**b**) MCF-7 cells treated with CMP−L−CA4 (50 nM), and (**c**) MCF-7 cells treated with a photosensitizer—core-modified porphyrin IY69 (5 μM). Irradiation with a 690 nm diode laser (11 mW/cm^2^ for 15 min) was performed only on the left side in each image. Reproduced with permission from [68].

**Figure 6:**
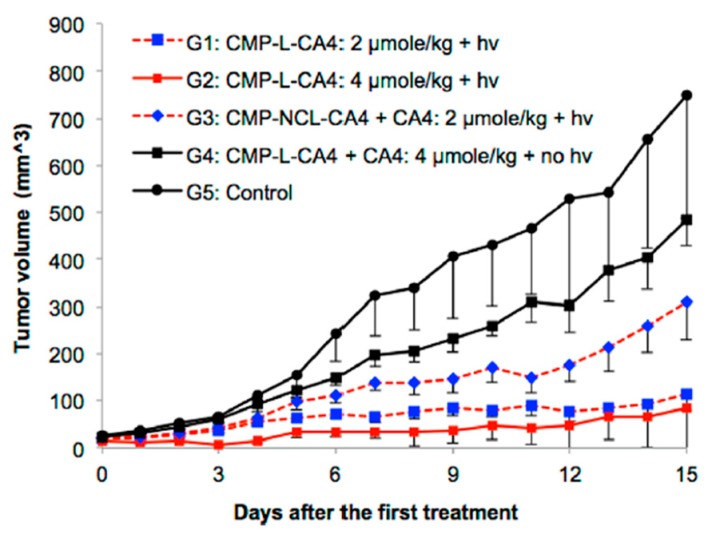
Antitumor effects of the CMP-L-CA4 prodrug-PDT system. Mice were injected with a formulation containing designated compounds once a day on days 0, 1, and 2; hν = 360 J/cm^2^ with 690 nm; and six mice were used per group, except for the control group, which used three mice. *p* < 0.05 between G2 and G4, between G3 and G5, and between G1 and G3. *p* > 0.05 between G4 and G5. Reproduced with permission from [68].

Subsequently, CA4 and a fluorescent probe were conjugated to a near-IR photosensitizer through the aminoacrylate linker; and the activation of the drug/fluorescent agent was tested in tubulin polymerization assay, on cytotoxicity assay, and in mice (Table 1, entry 5a). CA4 conjugated to the photosensitizer was unable to inhibit tubulin polymerization (6% inhibition as compared to 100% inhibition of CA4). There was a noticeably significant difference in cytotoxicity between CA4 conjugated to the photosensitizer through the singlet-oxygen-sensitive linker and CA4 conjugated through a noncleavable linker (Table 1, entry 5a, b). The IC_50_ of cleavable conjugate in the dark was 164 nM, compared with 1802 nM of the noncleavable mimetic. Nevertheless, a significant difference in cytotoxicity was observed between the two in the presence of light. Photocleavable conjugate showed a six-fold decrease in IC_50_ cytotoxicity value (from 164 nM to 28 nM), compared with only 1.7-fold of the noncleavable analog (1802 nM to 1063 nM) (Table 1). More importantly, in a cell-based assay, the CA4 prodrug-PDT system showed the significant effect of killing nearby cells, compared with PDT alone (Figure 5). In mice, the combination system showed only minimal effect without illumination, but produced enhanced antitumor effects once activated with light, e.g., *p* < 0.05 between group G2 and group G4 in Figure 6. No significant signs of acute toxicity were observed during the treatment with the prodrug-PDT system in mice models [68].

**Figure 7:**
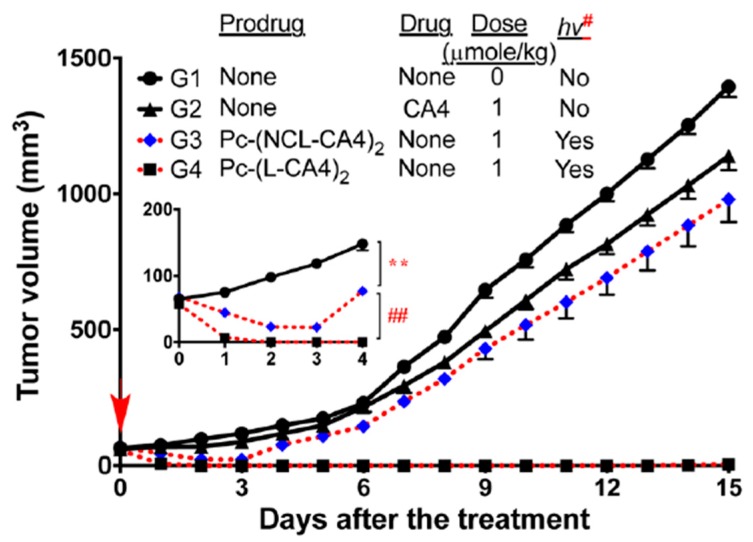
Antitumor effects of the Pc-(L-CA4)_2_ prodrug-PDT system. Drugs were intravenously (IV) administered once on day 1. Illumination was performed 24 h after drug administration (hv^#^: 100 mW/cm^2^ for 30 min (180 J/cm^2^) or 200 mW/cm^2^ for 30 min (360 J/cm^2^). ** *p* < 0.01 (G1vs. G3, from day 1 to day 4) and ## *p* < 0.01 (G3 vs. G4, from day 1 to day 4)). Reproduced with permission from [69].

The same drug (CA4) was conjugated to another photosensitizer that served as both singlet-oxygen-generator and fluorescent probe to determine an illumination time when the prodrug was at its optimal concentration at the target site. Silicon phthalocyanine (Sens/Fl, Table 1, entry 7) was chosen as a photosensitizer because it has a unique balance of both functionalities, i.e., quantum yield for singlet oxygen = 0.22 and fluorescence = 0.4 and a high molar extinction coefficient (ε = 150,000 M^−1^cm^−1^ at 675 nm) [69]. Two molecules of CA4 drug were linked to the sensitizer through either aminoacrylate linker (Table 1, entry 7a) or a noncleavable linker analog (Table 1, entry 7b) as a control. Regarding in vitro activity, both photocleavable and noncleavable compounds showed significantly lower inhibitory activity in a tubulin polymerization assay (23% and 17% for PDT-cleavable prodrug and noncleavable mimetic, respectively) compared with the parent drug CA4 (100%) at 3 μM concentration of each (*p* < 0.02 for both PDT-cleavable prodrug and noncleavable mimetic as compared to CA4 group). The toxicity IC_50_s against MCF-7 cells was determined to be 173 nM (PDT-cleavable prodrug) and 916 nM (noncleavable mimetic), compared with around 7 nM for CA4. Illumination restored the toxicity of the PDT-cleavable prodrug (IC_50_ = 6 nM). Significant cytotoxicity was also observed for the noncleavable prodrug with illumination, demonstrating the highly potent PDT effect of silicon phthalocyanine (IC_50_ = 34 nM). In mice, accumulation of fluorescence in tumors, reaching a maximum at around 24 h post injection, was observed when the animals received photocleavable prodrug or noncleavable analog (through polymer micelles formulation). Outstanding antitumor effects were observed in mice treated with PDT-cleavable prodrug, (1 μmol/kg + hν (100 mW/cm^2^)) or (2 μmol/kg + hν (200 mW/cm^2^)). Tumors shrank and remained nonmeasurable for almost 15 days in the PDT-cleavable prodrug and light treatment group, while tumor sizes reached > 800 mm^3^ after 12 days in the no-treatment control group. All mice treated with PDT-cleavable prodrug and light lived until day 30. Noncleavable mimetic and light treatment (1 μmol/kg + hν (100 mW/cm^2^)) had a temporary impact on tumor size only until day 3, and the tumors grew back at a rate similar to that of the tumors in the no-treatment group (Figure 7). No mice experienced a significant loss in body weight during study duration (15 days), suggesting minimal acute toxicity of the combination treatment. The combination system was also shown to provide better antitumor effects in cell-based and animal models, as the release of CA4 could overcome the spatiotemporal limitations of ^1^O_2_ in PDT alone [70].

**Figure 8:**
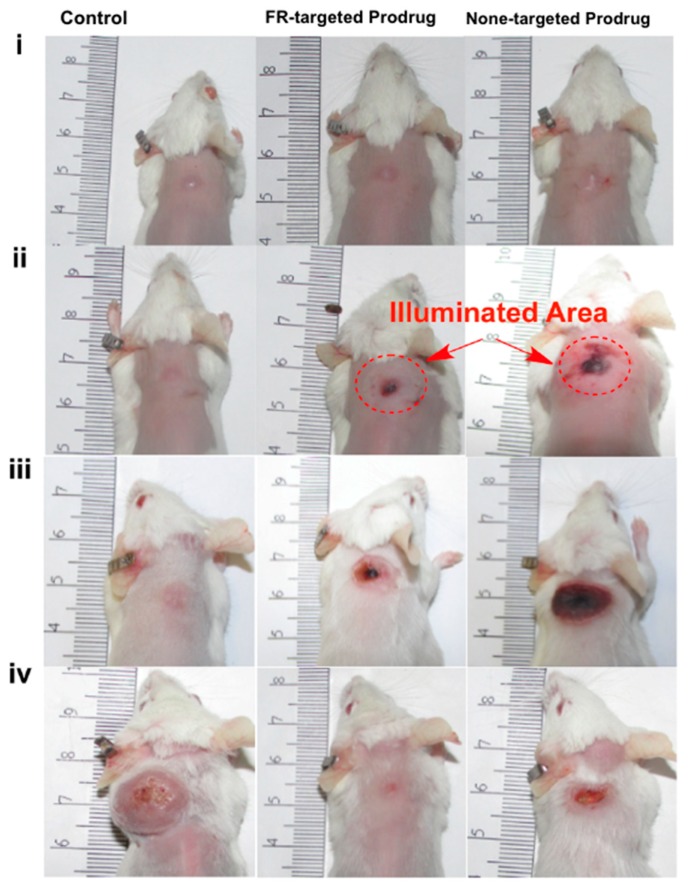
Selective tumor damage with minimal skin damage in a broad illuminated area of mice treated with a folate-receptor-targeted and nontargeted prodrug. Prodrug with a polyethylene glycol (PEG) length of ~45 in Table 1, entry 7 was used as an FA-targeted prodrug, and prodrug with PEG length of 18 units (nontargeted) in Table 1, entry 7 was used as a nontargeted prodrug. Prodrug dose was 2 µmol/kg, administered once. (**i**) Day 0 before illumination, (**ii**) day 1, (**iii**) day 6, and (**iv**) day 15 post illumination. Reproduced with permission from [71].

The effect of a tumor-targeting group on the PDT-activating CA4 prodrug was also tested in our lab. CA4 was connected to the silicon phthalocyanine core on one side through the aminoacrylate linker, while a folate (FA) moiety was attached to the sensitizer on the other side. A polyethylene glycol (PEG) spacer of various lengths was placed between the FA and Sens/Fl moiety (Table 1, entry 8). The folate receptor was targeted, as it was reported to be overexpressed in many tumors, such as ovarian, lung, colon, and breast cancers. Folate−drug conjugates have been developed and tested in cells, animal models, and human clinical trials with successful results. Additionally, PEG was introduced to help increase the solubility of the prodrugs, as they are highly hydrophobic and readily form aggregates in aqueous conditions. It was expected that PEG conjugation would reduce the aggregation and nonspecific uptake of the resulting conjugates by the cells or tumors, as well as increase the targeting capability of the construct. A cell-based assay showed that the FA-conjugated prodrugs were more potent than non-FA-conjugated analog. The PEG length affects solubility and cellular uptake of the prodrug, as well as its cytotoxicity upon activation. Upon activation with light, the cytotoxicity IC_50_ against colon 26 cell lines was determined to be 17, 27, 40, 45, and 49 nM for a PEG of 45, 18, 1, 0, and 18 units in length without the FA group, respectively (Table 1, entry 8). Among the constructs listed above, in mice, a targeted prodrug containing a PEG of ~45 units was delivered to tumors most selectively. Selective and effective tumor damage was observed with minimal skin damage in a broad illuminated area, suggesting the contribution of the targeting FA group (Figure 8) [71].

In addition to directly linking CA4 to a photosensitizer, Bio et al. investigated the capability of activating aminoacrylate-linked CA4 in an intermolecular fashion by localizing both prodrug and photosensitizer to the mitochondria (Table 1, entries 9 and 10). This approach offers the flexibility of reducing prodrug size, which can help improve prodrug penetration and potentially selectivity in damaging the tumor due to multiplying selectivity of the two components in the system. Colocalization of the prodrug and photosensitizer to the mitochondria helps bring those components close enough to each other to ensure efficient cleavage of the linker mediated by singlet oxygen. The CA4 prodrug was targeted to the mitochondria by conjugating it to a rhodamine group (Table 1, entry 9). The photosensitizer, protoporphyrin IX, was formed in the mitochondria of cells from a hexyl-5-aminolevulinate precursor. Using a fluorescent mimetic, rapid and sufficient (72%) release of the conjugated moiety was estimated in the presence of light (Table 1, entry 10). In rat bladder cancer cells (AY-27), administration of the photosensitizer precursor and photoactivatable rhodamine-conjugated CA4 prodrug (0.1–1.25 μM) in the presence of light showed almost 100% cell killing, while no dark toxicity or phototoxicity of the photoactivatable conjugate in the absence of photosensitizer was observed, up to the prodrug concentration of 1.25 μM. When only the photosensitizer precursor was administered without prodrug, only about 50% of cell killing was observed. The increase in cytotoxicity was attributed to the combination effect of CA4 and PDT, compared with PDT alone [73].

Paclitaxel is another drug that was investigated with our PDT activating system [11,72,74]. PTX was connected to the aminoacrylate linker by an ester bond with an alcoholic hydroxyl group of PTX (Table 1, entry 7). Thapa et al. conjugated two PTX molecules to a silicon phthalocyanine moiety in a manner similar to that of a CA4 prodrug (Table 1, entries 6 and 8), using a facile synthesis, mild reaction conditions, and moderate yields. The PTX prodrug was shown to be stable in the dark in the presence of cells. An intact PTX molecule was rapidly released upon illumination of the prodrug (93% PTX was released under 30 min illumination at 690-nm laser light and 5.6 mW/cm^2^). Activity of PTX was abolished when it was conjugated to the sensitizer, as demonstrated by tubulin polymerization assay and cell-based toxicity assay (toxicity IC_50_ against SKOV-3 cell lines was 910 nM, compared with 5 nM of PTX, a 190-fold reduction in toxicity). Once illuminated, the prodrug showed a strong cytotoxicity with an IC_50_ of about 4 nM—comparable to that of PTX.

Thapa et al. further investigated the effect of PEG conjugation and folate receptor targeting on PTX prodrugs. Using a design similar to that of one of the CA4 prodrugs, PTX was connected to a silicon phthalocyanine on one side, and a folic acid (FA) moiety was attached to the sensitizer on the other side through a PEG of various lengths (~0, ~23, ~45, ~80, and ~114 PEG units) (Table 1, entries 7 and 11). Similar to what was observed with PEG-conjugated CA4 prodrugs, the PEG length affected nonspecific and folate-receptor-mediated uptake of the prodrugs. Prodrugs with medium-sized PEGs (23, 45, and 80 PEG units) showed enhanced cellular uptake in folate-receptor-positive SKOV-3 cells, as compared with targeted prodrugs with the longest PEG (114 PEG units) and with no PEG (12-fold increase in the intracellular prodrug accumulation at 24 h over that of no-PEG prodrug). In the dark, low toxicity was observed for all folate–PEG-conjugated PTX prodrugs, as demonstrated by >90% survival of SKOV-3 cells after 72 h of incubation with prodrugs at a concentration of 500 nM. In the presence of light, medium-sized FA–PEG-conjugated prodrugs showed more potent photocytotoxicity (IC_50_s around 130 nM) than prodrugs with no PEG or longer PEG (114 PEG units, IC_50_ ∼400 nM) [74].

PTX prodrug activated by PDT in an intermolecular system was also investigated. Bio et al. investigated conjugation of PTX to three different mitochondriotropic lipophilic cations, rhodamine, 4-carboxy-1-methylpyridinium chloride (CAT), and triphenylphosphonium (TPP), using photoactivatable aminoacrylate linker (Table 1, entry 12). The prodrugs were expected to accumulate in the mitochondria of cells and to be activated with light when the hexyl-5-aminolevulinate (HAL) precursor was administered. In a cell-based study, at a prodrug concentration of 1.25 μM with HAL, no significant cytotoxicity was observed in the absence of light. Upon illumination, however, 84%, 82%, and 80% of cells were killed with 0.25 μM rhodamine-, TPP-, and CAT-conjugated PTX prodrug, respectively. Under the same conditions, PDT alone killed only about 40% of the cells, suggesting the enhanced cell killing effect of the PDT and singlet-oxygen-activatable prodrug combination system [11].

In the animal studies with our prodrugs, we did not observe any significant chronic toxicity monitored by visual observation and body weight change. That is because animals were treated only once in our standard treatment condition. However, more systemic study for chronic toxicity remains to be performed.

## 4. Challenges for Clinical Translation of Prodrug

Lack of efficacy is one of the top reasons for late-stage clinical failure of drug development [75]. This causes an incredible waste of time and money invested on early drug development. There is a missing link between early drug design and clinical outcome. Quantitative systems pharmacology (QSP) is an emerging field that investigates the relationship between drugs, biological systems, and the disease process using a computational and modeling technique on multiple scales from the molecule, organ, and rodent to patients [76]. QSP has been applied to many early drug developments to improve the probability of clinical success [77,78,79].

There are many kinetic processes involved in prodrug delivery. Several things need to be considered for our prodrug efficacy: 1. Once it goes into systemic circulation, it will go through elimination by the liver or kidney. With pegylated modification of the structure, it may be trapped in the reticuloendothelial system (RES), which prolongs its systemic circulation time [80]. However, pegylation can also prevent the prodrug from undergoing cell membrane binding and endocytosis; 2. Tumor vasculature and cancer cells are both potential targets of PDT damage. To target the cancer cell, our prodrug will be transported to the tumor via blood circulation. It will reach the individual cancer cell by extravasation into the tumor interstitium. Meanwhile, the concentration of the prodrug remaining in the tumor vasculature will decline due to systemic clearance. This will cause a loss of vasculature damage from PDT [81]; 3. The released anticancer agent will either bind to the intracellular target or diffuse to neighboring cells. As we can see here, all these processes produce opposite outcomes. It is critical to have a quantitative guideline to start an optimal experiment design. As such, QSP is an ideal tool to investigate the complexity of the system.

## 5. In Vitro Efficacy Evaluation of Prodrugs Guided by Quantitative Systems Pharmacology (QSP)

**Figure 9:**
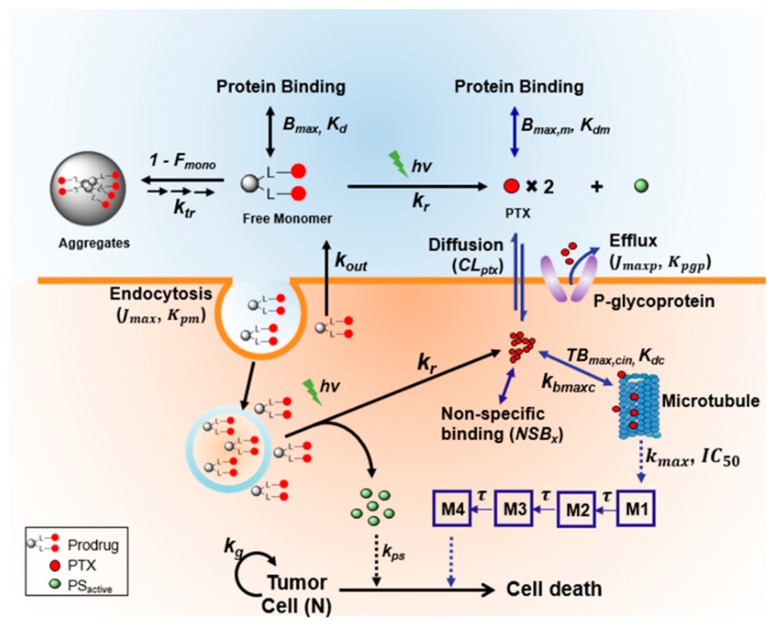
Schematic diagram of the intracellular trafficking and dynamic model for light-responsive prodrug. After 24 h incubation of SKOV-3 cells with Pc-(L-PTX)_2_, the cells were illuminated using a 690-nm diode laser (hv) at 5.6 mW/cm^2^ for 30 min. The model components describing the kinetic and dynamic processes of the released paclitaxel (PTX) (blue arrows) only apply to the cleavable Pc-(L-PTX)_2_. Reproduced with permission from [82].

In our lab, we chose the Pc-(L-PTX)_2_ (entry 8) prodrug to begin our in vitro investigation based on a QSP approach (Figure 9) [82]. We found that around 80% of our prodrug formed aggregates in medium, due to its high lipophilicity. Only 20% of free prodrug can be internalized into cells. However, disaggregation of Pc-(L-PTX)_2_ was observed 9 h after incubation, which led to a higher intracellular accumulation of Pc-(L-PTX)_2_ than observed with a control Pc-(NCL-PTX)_2_ (a noncleavable mimetic of Pc-(L-PTX)_2_) at 24 h. After 24 h incubation with prodrug, the cells were exposed to light illumination using a diode laser (690 nm) at 5.6 mW/cm^2^ for 30 min. Three days after illumination, MTT assays were performed to evaluate PDT damage alone and PDT damage combined with PTX damage from prodrug.

We found that our prodrug was more potent than PDT treatment alone (IC_50_ = 3.9 vs. 24 nM) due to released PTX [72]. This is what we called the bystander effect by the site-specific released anticancer drugs [83]. However, the in vitro results cannot always represent what happens in vivo. There were numerous discrepancies between the in vitro and in vivo antitumor effects [84]. To get a better insight into in vivo conditions, we replaced the medium immediately after illumination to mimic the in vivo condition of blood flow, a route for clearance of locally released drugs. The cytotoxicity of Pc-(L-PTX)_2_ was largely diminished when we replaced the medium. In order to gain a systemic insight, we developed a quantitative mathematical model describing the intracellular trafficking of the prodrug and released drug. We used our developed model to evaluate the influence of each factor on the antitumor effects (in silico experiment) and to identify the key determinants of overall drug efficacy. We identified the following key highlights: 1. PDT effect is determined mainly by prodrug intracellular accumulation; 2. Bystander effect from released PTX is mostly influenced by its retention in the local tumor area; 3. Target-binding affinity of the released anticancer drug is critical for anticancer efficacy. In this study, PTX was the linked anti-cancer agent. PTX is known to act by binding to β-tubulin in assembled microtubules and stabilizing them against disassembly. Based on our model simulation, its target binding affinity can significantly influence the prodrug efficacy in vivo. Among all taxane analogs, PTX showed a higher binding ability towards microtubules [85,86]. This result helped us to select the anticancer agent (PTX) for the following in vivo studies. The model can also be applied to other categories of anticancer agent selection.

## 6. PK Properties of the Prodrug

Several studies have reported the pharmacokinetic (PK) characteristics of PS itself [87]. There is a wide variation in PK profiles of different PSs. For example, Photofrin is reported to have a long-term half-life (156 days) in patients, while TOOKAD^®^ undergoes a rapid elimination with a half-life of 1.3 h [88,89]. In most cases, PSs were found to accumulate in liver [90,91]. PS also accumulates in the spleen, lung, and kidney in several studies [92,93].

So far, only a few studies have investigated the PK of singlet-oxygen-activatable conjugates [94]. By adding the linker and anticancer drug, changes in structure, molecular weight, and physical characteristics may cause the alteration in PK profiles of photosensitizer conjugates. Furthermore, the anticancer drug is delivered through the PS vesicle. This can further change the PK of anticancer drugs in comparison to conventional administration.

The different PK profiles will impact the selection of light illumination time. PDT damages tumors via three well-known mechanisms: Direct cancer cell killing, vascular damage, and immune activation [95]. Prodrug accumulation between cancer cells and tumor vasculature varies based on its PK profiles. Different PK profiles will influence the time of light delivery selection: Drug-light interval (DLI), which is important for PDT mechanism and antitumor effect. The criteria for DLI selection are largely dependent on PK properties (PS accumulation in tumor, blood PS concentration, etc.). Therefore, it is important to understand prodrug PK before we start any treatment. 

We chose FA–PEG_45_–Pc-L-PTX to perform PK investigation because it showed the highest cancer cell accumulation in vitro [74]. The PK studies were performed in mice with an intravenous bolus administration of 2 µmol/kg prodrug [96]. The plasma half-life of the prodrug was 8.6 h, which is longer than that of free PTX (T_1/2_ = 1.96 h). The longer remaining time in the vasculature gave us a flexible range to choose a DLI that could cause vascular damage. To add, we found a high accumulation of our prodrug in the tumor area due to the target delivery. The tumor concentration (>2 µM) was kept above IC_50_ from 12 to 48 h after prodrug administration [74]. Prodrug also accumulated in the liver, spleen, and kidney, which are the major organs for metabolism.

## 7. Treatment Optimization Aided by Physiologically Based Pharmacokinetic Modeling

**Figure 10:**
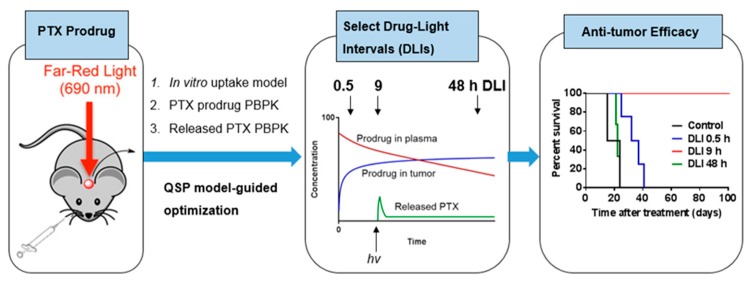
In vivo optimization of PTX prodrug treatment. The mice were intravenously administered PTX prodrug. The drug light intervals (DLIs) were selected based on prodrug PK profile and PBPK model-predicted released PTX profile. The PTX prodrug was illuminated at selected DLIs for antitumor efficacy evaluation. Produced with permission from [96].

With an in-depth understanding about significant events for prodrugs, released drugs, and key determinant variables in in vitro conditions, we further modified the prodrug for better in vivo efficacy (Figure 10). First, to achieve a higher and more selective accumulation in the tumor, we added a tumor-targeting group (folic acid, FA) and a spacer of PEG to our PTX prodrug [74]. According to our in vitro results, the modified PTX prodrug demonstrated an over 15-fold increase of uptake and 70% folate-receptor-mediated uptake [74]. While PTX is a highly lipophilic small molecule with a logP of 3.96 and molecular weight (MW) of 854 g/mole, the prodrug is much larger and more hydrophilic, with a logD of 7.4 and MW of ~4226 g/mol, due to the PEG moiety (for a PEG of 45 units in length) [74]. Due to its different physical characteristics, the in vivo PK study showed that PTX prodrug had a longer circulation half-life (T_1/2_ = 8.6 h vs. 1.96 h (PTX)) and restricted volume of distribution (Vss = 0.14 L/kg vs. 6.69 L/kg (PTX)) compared with free PTX [97].

PDT damages tumors via three well-known mechanisms: Direct cancer cell killing, vascular damage, and immune activation [95]. Direct cancer cell killing and vascular damage play an important role in immediate damage, while immune activation also contributes to sustained tumor damage [34]. Whether direct killing or vascular damage is more dominant depends on where the photosensitizer remains when illumination is made [98]. Distribution of photosensitizer in the tumor vasculature causes more vascular damage, while accumulation in cancer cells is better for direct killing. Based on our prodrug PK, we found that most of the prodrug remained in the plasma at an early time point (<1 h) and accumulated in the tumor after 9 h. We selected three different drug light intervals (DLIs = 0.5 h, 9 h, and 48 h) to test its antitumor efficacy [96].

**Figure 11:**
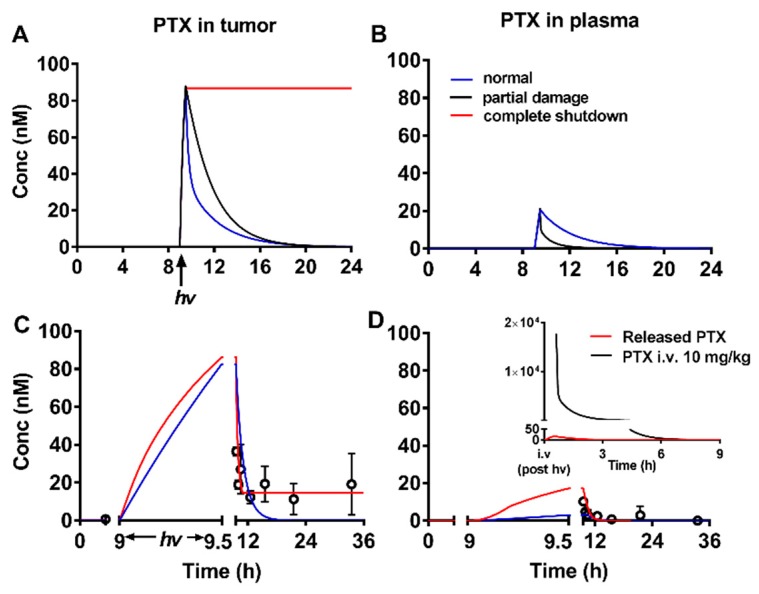
(**A**) Predicted time–concentration profile of PTX release in tumor; (**B**) predicted time–concentration profile of PTX release in plasma. With complete shutdown, no PTX is released in plasma; (**C**) simulated time–concentration profile of released PTX in tumor during/post illumination; (**D**) Simulated time–concentration profile of released PTX in plasma during/post illumination. Reproduced with permission from [96].

In this study, PTX was delivered through our prodrug. To predict the amount of PTX released in the tumor with different DLIs, we built a physiologically based pharmacokinetic (PBPK) model to describe (1) tissue distribution of the PTX prodrug, (2) intracellular delivery of the PTX prodrug, and (3) biodistribution of released PTX [96]. As we mentioned above, PTX was delivered and released in the tumor through the prodrug system. Therefore, the released PTX PK profile was different to that of free PTX. We applied our PBPK model to predict released PTX kinetics in the tumor and plasma (Figure 11). Because blood flow to the tumor (Q_T_) was influenced by antivascular effects from PDT, we simulated various released PTX kinetics profiles with different Q_T_ values. Interestingly, we found that the retention of released PTX was significantly impacted by Q_T_ values. PTX was quickly cleared from the tumor under a normal blood flow rate. However, PTX could remain in the tumor for over 24 h if the tumor capillary was completely blocked. Based on the simulation results, we hypothesized that the combination effect of PDT and PTX could be enhanced by optimized DLIs. We selected three different DLIs (0.5, 9, and 48 h) based on the prodrug PK profile to test its anticancer efficacy. The in vivo results demonstrated that the 9 h DLI had the best anticancer efficacy. At 9 h DLI, there was a balanced distribution of prodrug in the plasma and tumor, which could lead to both direct cancer cell killing and vascular damage. The damaged vasculature in the tumor area slowed the blood/tumor flow rate, which helped the PTX to remain in the tumor area [96].

With the in-depth understanding of prodrug PK and the help of advanced mathematical models, we can better design our future study. There will be a greater possibility to translate our preclinical results to clinical applications.

## 8. Conclusions

In recent years, significant progress has been made in developing advanced combination therapies involving PDT and chemotherapy without major systemic side effects. Our unique PDT (i.e., singlet-oxygen)-activatable prodrug system has demonstrated promise. Aminoarylate linker was readily cleaved by illumination on the photosensitizer both in vitro and in vivo. QSP has become an essential tool for us to understand the pharmacokinetics and pharmacodynamics of the prodrug and to optimize the efficacy of the prodrug in preclinical models. We were able to demonstrate effective and long-term control of local tumors using small and large tumor models under the optimized conditions. Currently, we are advancing our singlet-oxygen-activatable prodrug strategy in various angles, such as broader adaptability of functional groups for the singlet-oxygen-cleavable linker, bimolecular activation, QSP modeling incorporating more pharmacodynamics, tuning mechanism and pharmacology of prodrugs, and clinical translation.

## Figures and Tables

**Table 1:** List of drugs, singlet-oxygen-activatable prodrugs, and fluorescent probes using aminoacrylate linker; cytotoxicity (IC_50_) values.

Entry	Drug	Prodrug Structure (Name)	Wavelength; IC_50_s (nM) without vs. with hv; Cell Line	Ref.
1	Estrone	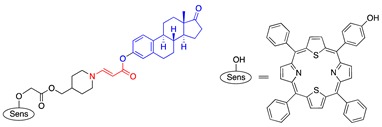	690 nm	[66]
2	SN-38	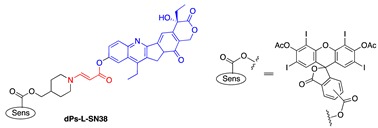	540 nm; 820 vs. 218; MCF-7	[67]
3	CA4	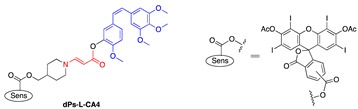	540 nm; 116 vs. 13; MCF-7	[67]
4	Coumarin	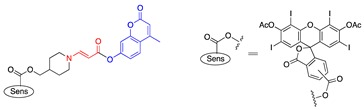	540 nm	[67]
5	CA4	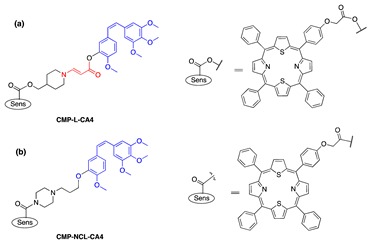	690 nm; (a) 116 vs. 13; MCF-7 (b) 1802 vs.1063; MCF-7	[68]
6	CA4	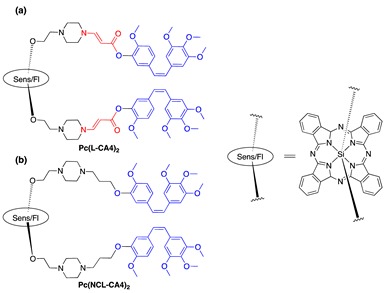	690 nm (a) 173 vs. 6; MCF-7 (b) 916 vs. 34; MCF-7	[69,70]
7	CA4	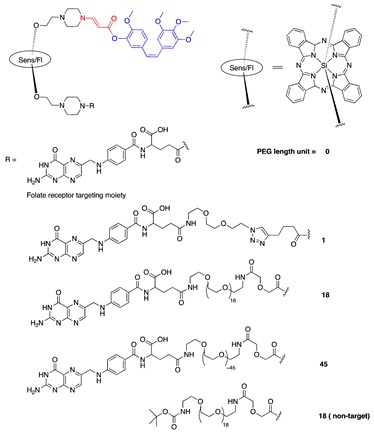	690 nm	[71]
8	PTX	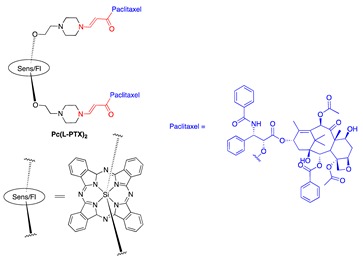	690 nm; 910 vs. 4; SKOV-3	[72]
9	CA4	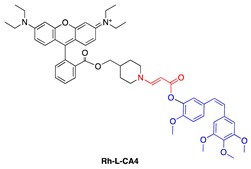	531 nm; ~100% survival up to 1.25 μM with HAL (hexyl-5-aminolevulinate, 0.5 mM) vs. 5% survial at 0.1 μM with HAL (0.5 mM)	[73]
10	BODIPY	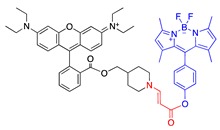	531 nm	[73]
11	PTX	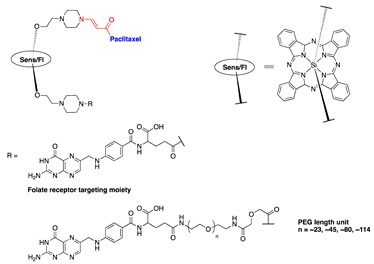	690 nm	[11,74]
12	PTX	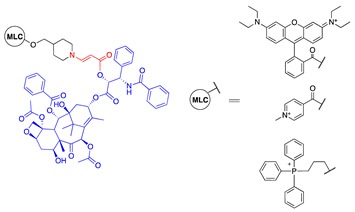		[11]
